# Supplementary material for: Spared perilesional V1 activity underlies training-induced recovery of luminance detection sensitivity in cortically-blind patients
Source: Nat Commun. 2021 Oct 20;12:6102. doi: 10.1038/s41467-021-26345-1 (PMC8528839; doi:10.1038/s41467-021-26345-1)
Supplement: Supplementary file 1 — Supplementary Information [file 41467_2021_26345_MOESM1_ESM.pdf]

Supplementary Figures

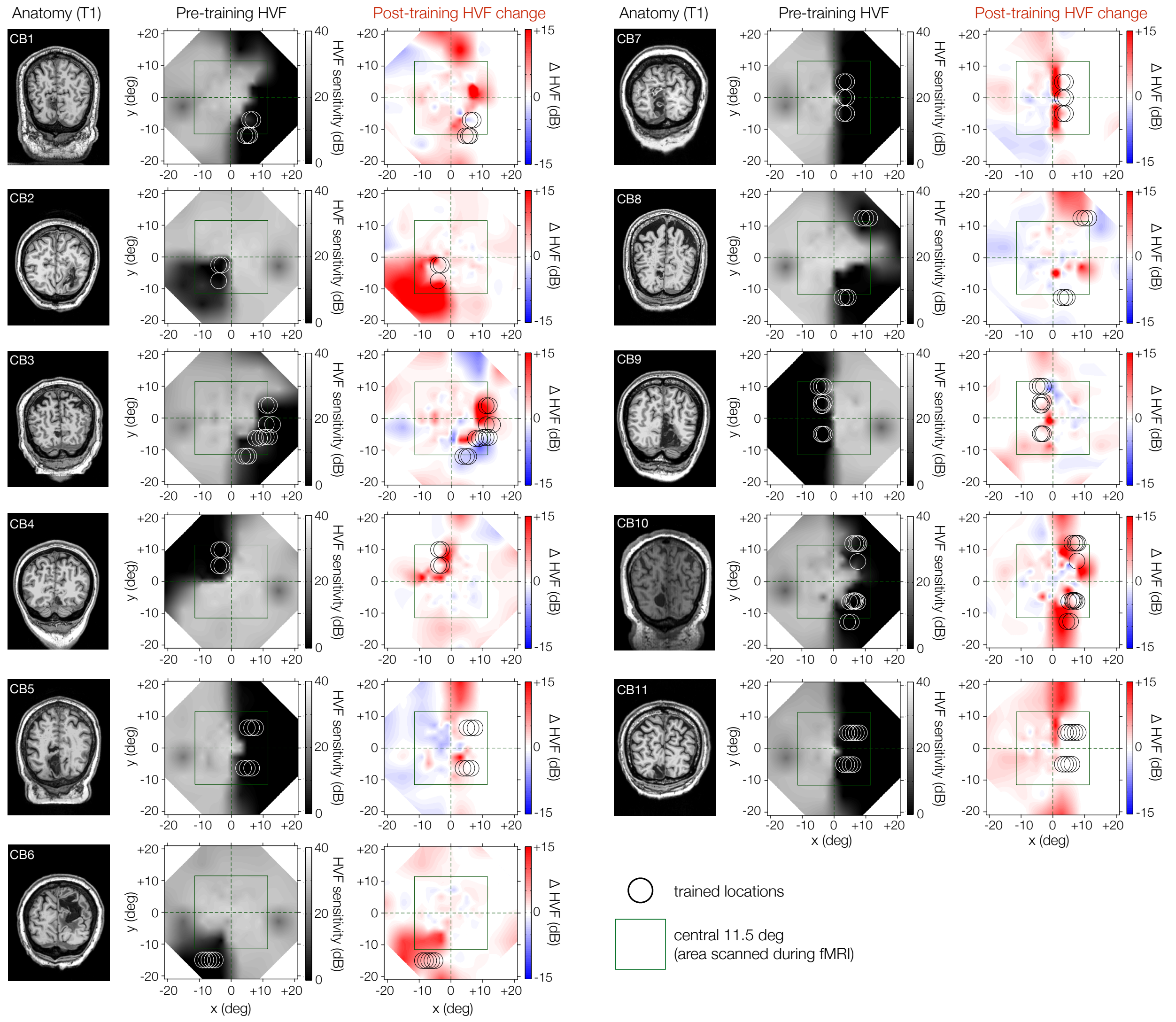

Figure S1. *Visual discrimination training results in recovery of conscious luminance detection sensitivity within the blind field of chronic CB patients.* T1-weighted images and corresponding baseline (pre-training) Humphrey Visual Fields (HVF; luminance detection sensitivity in dB) for all 11 chronic CB patients. Prior to training, all CB patients showed loss of HVF sensitivity (dark regions) within parts of their visual field. Following training, all patients showed improved HVF sensitivity (indicated in red) with very little worsening. Such recovery is not observed in untrained controls (Cavanaugh & Huxlin, 2017). Circles on the HVF maps indicate visual discrimination training locations for each CB patient. See the Discussion and our previous study in a larger cohort of trained and untrained chronic CB patients (Cavanaugh & Huxlin, 2017) for more details regarding the link between visual discrimination training locations and training-induced HVF recovery.

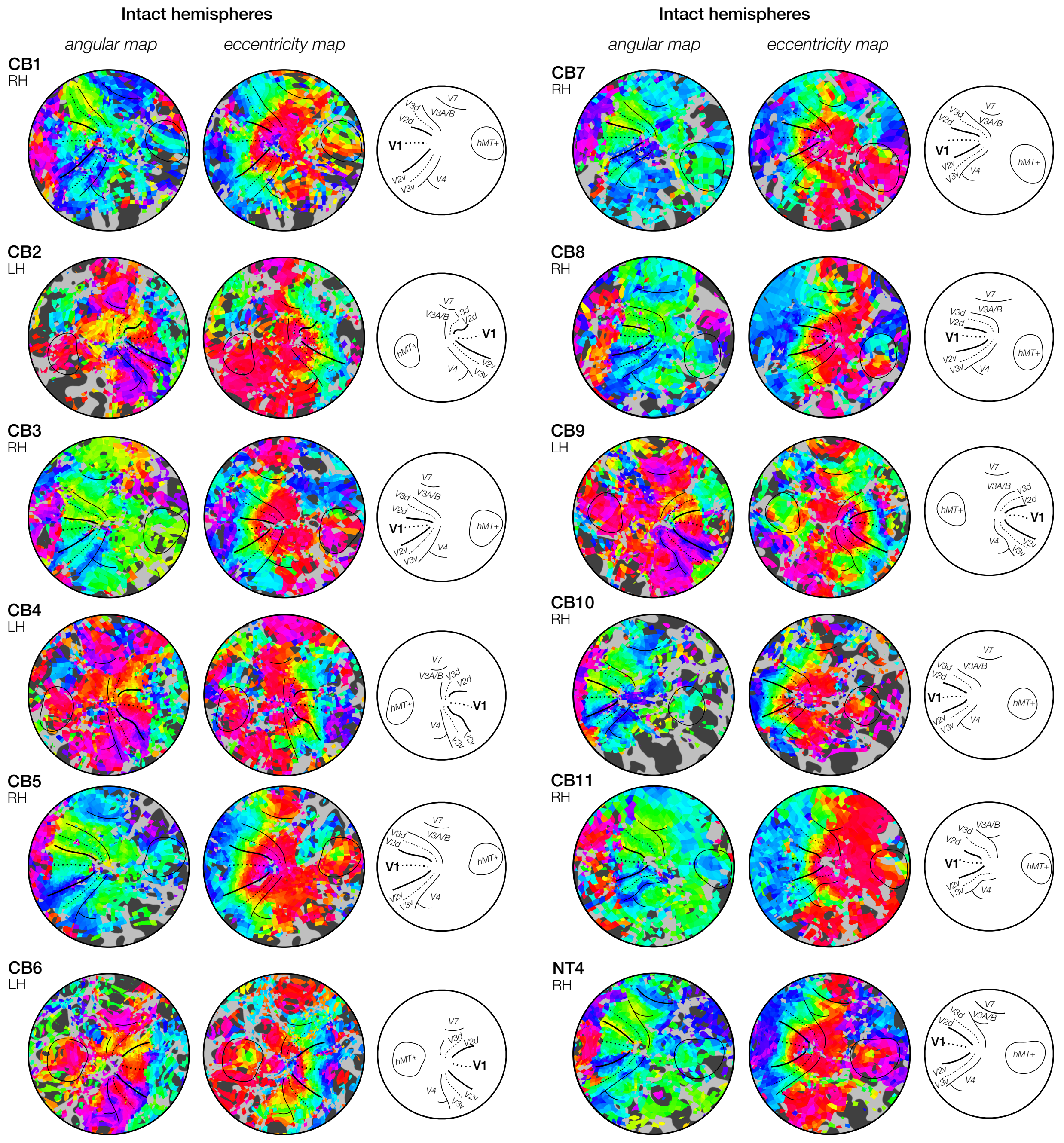

Figure S2. *Pre-training retinotopic maps of the intact hemispheres of all 11 chronic CB patients and of one visually-intact control.* Visually-evoked activity in the intact hemispheres of all 11 chronic CB patients is retinotopically organized and qualitatively similar to visually-intact controls, both in terms of radial and eccentric representations.

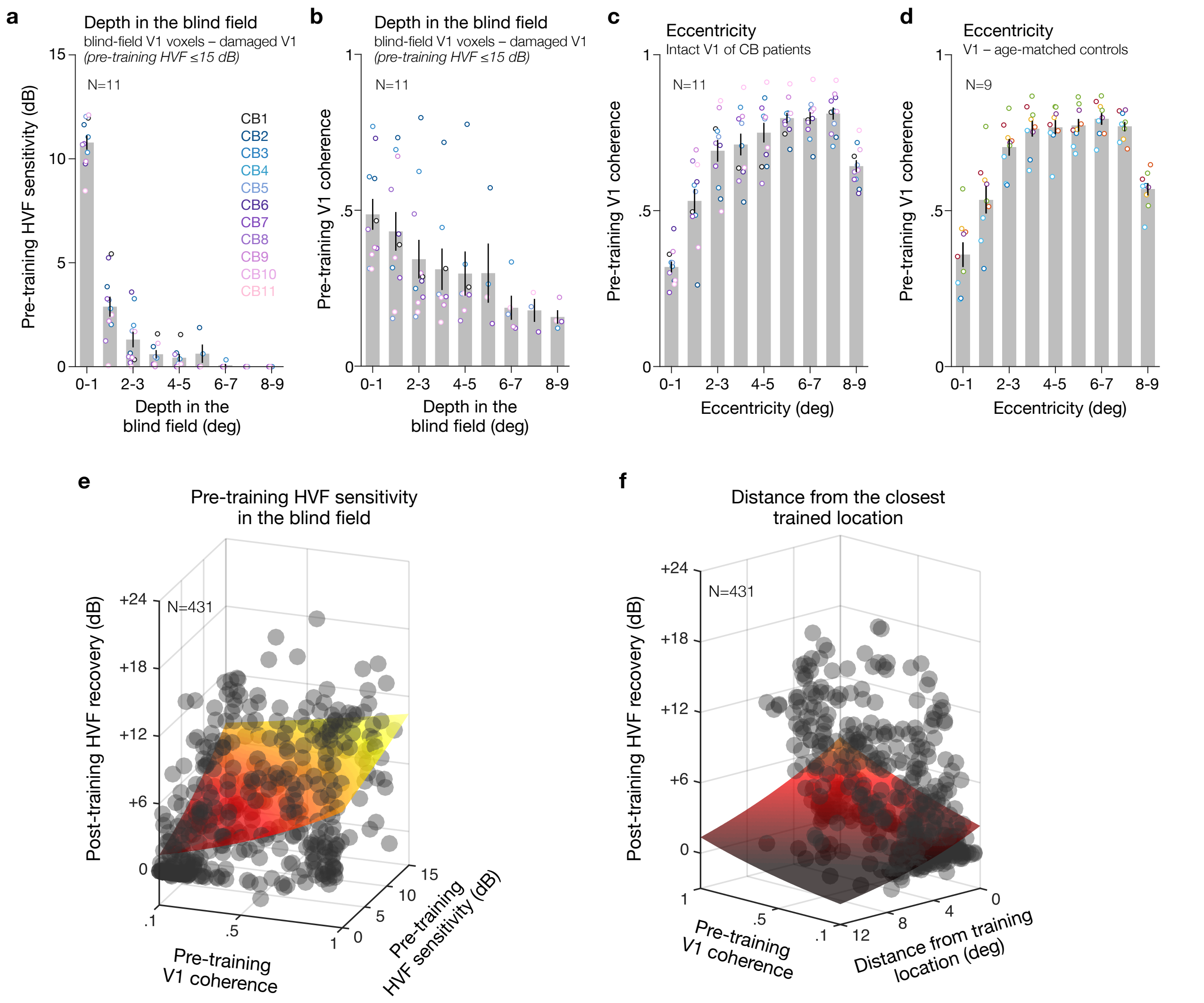

Figure S3. *Spared pre-training V1 activity predicts post-training Humphrey's Visual Field (HVF) recovery in the blind field of chronic CB patients.* **(a)** As expected, pre-training HVF sensitivity dropped rapidly with the depth in the blind field (computed as the distance from the blind-field border in deg). Bars represent average estimates across CB patients (±1SEM), with individual dots corresponding to individual CB patients. **(b)** Pre-training V1 coherence decreased as a function of the depth in the blind field. **(c,d)** Increased eccentricity is not associated with a drop in V1 response coherence, as observed in the intact V1 of CB patients (c, N=11) or in the intact V1 of age-matched control participants (d; N=9). **(e)** The strength of pre-training V1 responses and the pre-training HVF sensitivity at locations of the blind field were predictive of the magnitude of post-training HVF recovery, consistent with the link between the depth in the blind field and the drop in HVF sensitivity. **(f)** Although the strength of pre-training V1 responses was predictive of the magnitude of post-training HVF recovery, the distance from the closest trained location was not a good predictor of HVF recovery. Each data point corresponds to a V1 voxel, which were fit using a generalized linear mixed-effects model with participants as a random effect (colored surface).

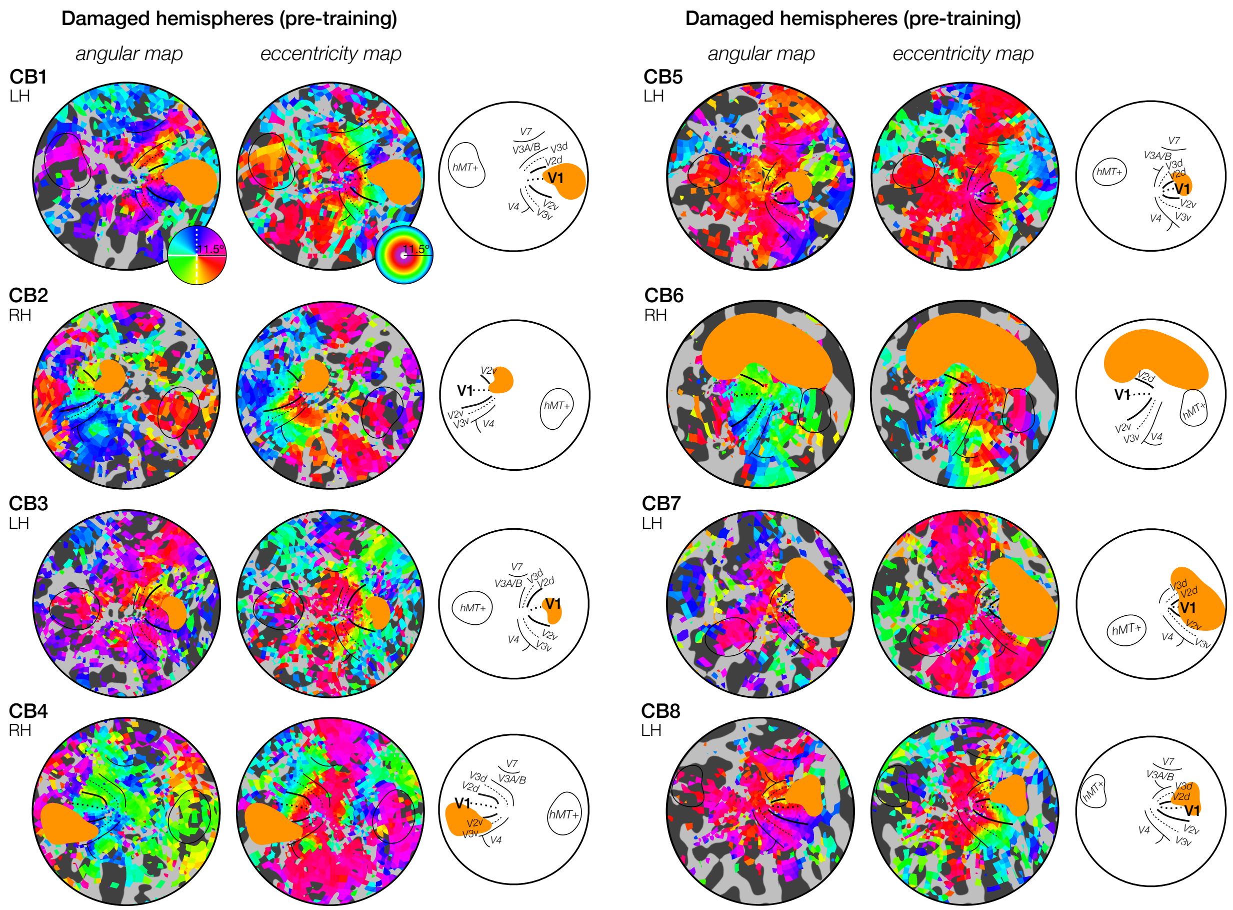

Figure S4. *Post-training retinotopic maps of the damaged hemispheres of the 8 chronic CB patients scanned during the post-training visit.* No coarse change in retinotopic organization was observed following training.

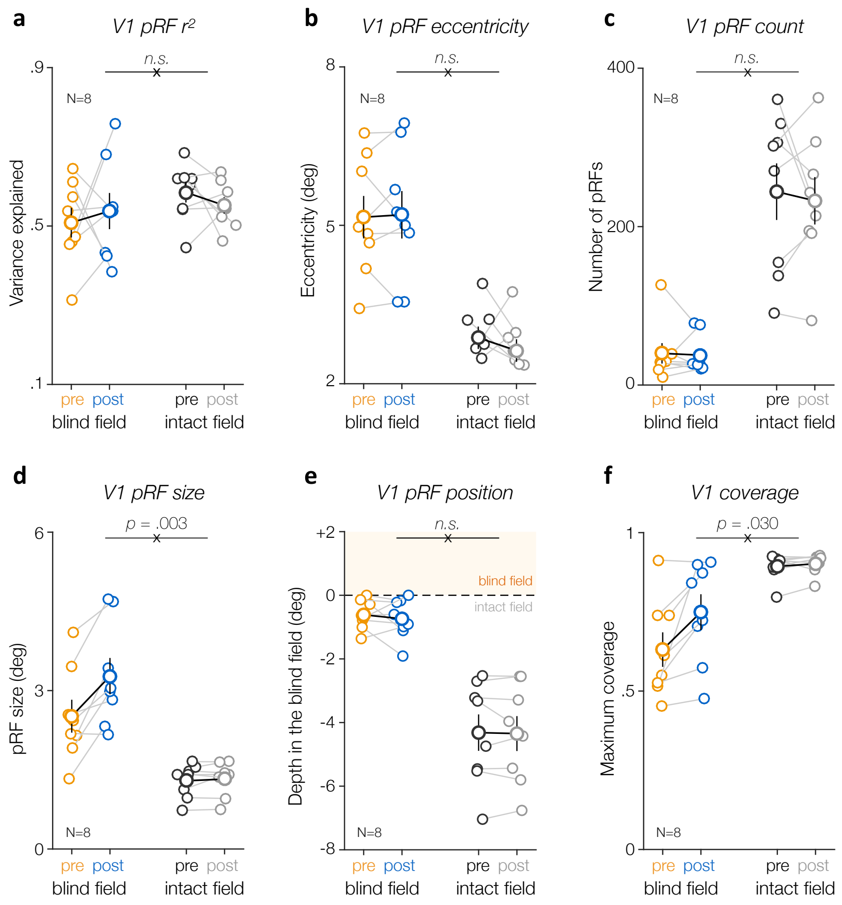

Figure S5. *Training-induced changes in V1 population receptive fields (pRFs) in CB patients (N=8).* **(a)** No difference in variance explained (r^2) was observed with training or between pRFs covering blind-field or intact-field regions. **(b)** pRFs covering blind-field regions were more eccentric than pRFs covering solely intact regions, as expected, with no effect of training. **(c)** Training was not associated with a change in the number of pRFs covering blind-field regions. **(d)** Training was associated with a significant increase in the size of V1 pRFs covering the blind field. **(e)** Training did not result in a change in pRF distribution around the blind-field border. **(f)** The increase in pRF size near the blind field border, without consistent changes in pRF number or position, resulted in a significant increase in visual-field coverage of the blind field in CB patients. Statistical values correspond to the training*visual-field coverage interaction. Smaller symbols with thin gray lines correspond to individual patients, with larger symbols with thicker black lines corresponding to group average values (±1 SEM).

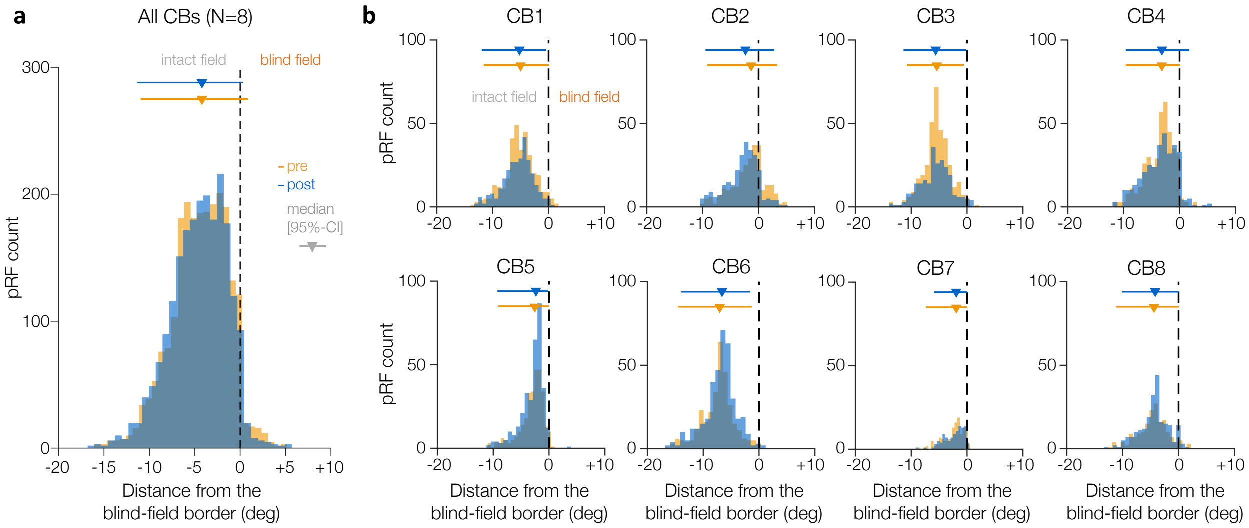

Figure S6. *Histograms of pRF position relative to the blind field border.* **(a)** pRFs combined across all CB patients (N=8) who were tested pre- and post-training. **(b)** Each of the 8 CB patients. No consistent change in pRF preferred position relative to the blind field border (depth in the blind field, in deg) was observed between pre- and post-training sessions. The vertical dashed line indicates the blind-field border, with the triangles and horizontal lines indicating the median and 95%-confidence interval for each training condition.

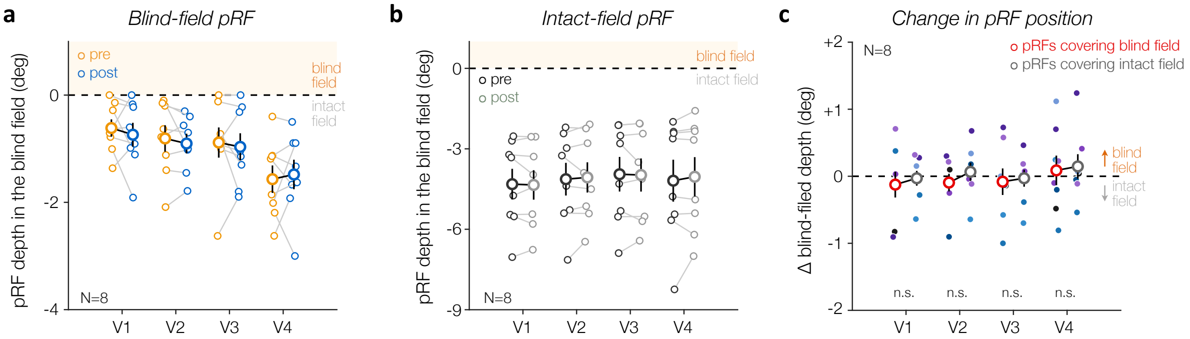

Figure S7. *No change in pRF position relative to the blind field border in V1-V4 following training of CB patients (N=8)*. **(a,b)** No clear difference in the position depth in the blind field (in deg) of pRFs covering either (a) blind-field or (b) intact-field regions. Note the difference in the y-axis scale of panels a and b. **(c)** Difference in blind-field depth (post-training *minus* pre-training, in deg) as a function of visual-field coverage (blind field or intact field) and visual areas (V1-V4). Same convention as Figure 8.

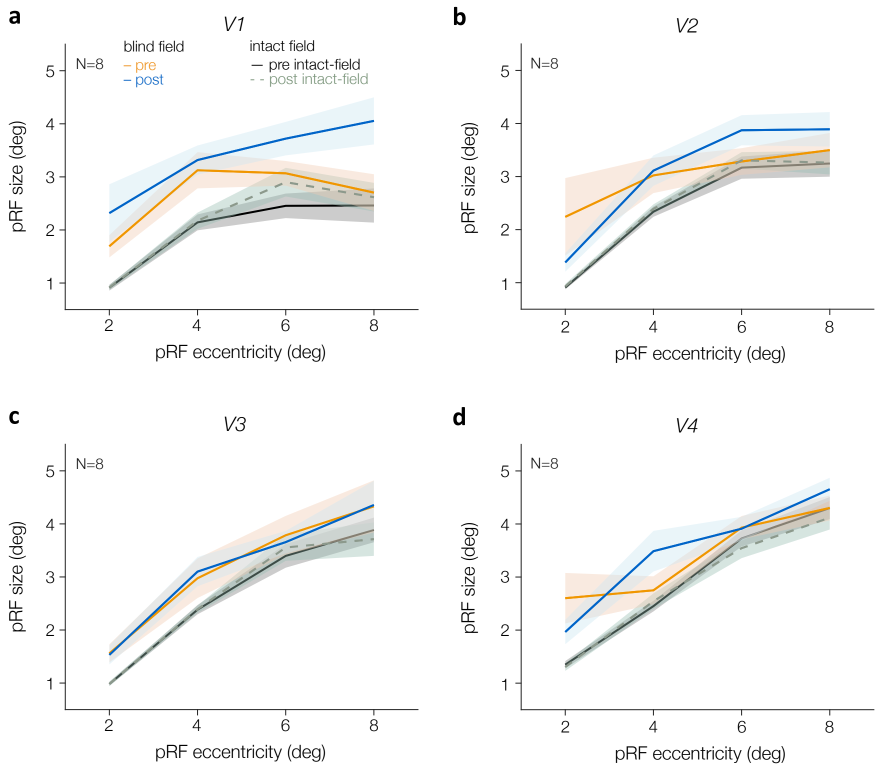

Figure S8. *pRF size as a function of pRF eccentricity for V1-V4 of trained CB patients (N=8).* Group-average pRF size computed using ±1 deg bins at 2, 4, 6 and 8 deg eccentricity, as a function of training and pRF coverage (blind field *vs* intact field). pRFs covering blind-field regions were larger than pRFs covering solely intact-field regions prior to training, and increased in size following training. These lesion-induced and training-induced patterns were pronounced for V1, but less clear in extrastriate areas (V2-V4).
