# Supplementary material for: Spared perilesional V1 activity underlies training-induced recovery of luminance detection sensitivity in cortically-blind patients
Source: Nat Commun. 2021 Oct 20;12:6102. doi: 10.1038/s41467-021-26345-1 (PMC8528839; doi:10.1038/s41467-021-26345-1)
Supplement: Supplementary file 3 — Reporting Summary [file 41467_2021_26345_MOESM3_ESM.pdf]

nature research

Corresponding author(s): Prof Krystel Huxlin

Last updated by author(s): Sep 3, 2021

Reporting Summary

Nature Research wishes to improve the reproducibility of the work that we publish. This form provides structure for consistency and transparency in reporting. For further information on Nature Research policies, see our Editorial Policies and the Editorial Policy Checklist.

Statistics

For all statistical analyses, confirm that the following items are present in the figure legend, table legend, main text, or Methods section.

n/a	Confirmed	
☐	☒	The exact sample size (*n*) for each experimental group/condition, given as a discrete number and unit of measurement
☐	☒	A statement on whether measurements were taken from distinct samples or whether the same sample was measured repeatedly
☐	☒	The statistical test(s) used AND whether they are one- or two-sided<br>*Only common tests should be described solely by name; describe more complex techniques in the Methods section.*
☐	☒	A description of all covariates tested
☐	☒	A description of any assumptions or corrections, such as tests of normality and adjustment for multiple comparisons
☐	☒	A full description of the statistical parameters including central tendency (e.g. means) or other basic estimates (e.g. regression coefficient) AND variation (e.g. standard deviation) or associated estimates of uncertainty (e.g. confidence intervals)
☐	☒	For null hypothesis testing, the test statistic (e.g. *F*, *t*, *r*) with confidence intervals, effect sizes, degrees of freedom and *P* value noted<br>*Give P values as exact values whenever suitable.*
☒	☐	For Bayesian analysis, information on the choice of priors and Markov chain Monte Carlo settings
☒	☐	For hierarchical and complex designs, identification of the appropriate level for tests and full reporting of outcomes
☐	☒	Estimates of effect sizes (e.g. Cohen's *d*, Pearson's *r*), indicating how they were calculated

Our web collection on statistics for biologists contains articles on many of the points above.

Software and code

Policy information about availability of computer code

Data collection	fMRI data was collected on a 3T Magnetom Trio scanner (Siemens, Erlangen, Germany) using MATLAB (R2015b, https://www.mathworks.com/products/matlab.html) and the MGL Toolbox (version 1.5; http://gru.stanford.edu/doku.php/mgl/overview) for stimulus presentation. Humphrey visual fields were collected using a Humphrey Field Analyzer (HFA II 750) by the same ophthalmic technician at the University of Rochester Medical Center.
Data analysis	fMRI data analyses were performed in MATLAB (R2015b, https://www.mathworks.com/products/matlab.html) using the open source software packages mrTools (version 3.5, http://gru.stanford.edu/doku.php/mrtools/overview) and FreeSurfer (version 5.3, http://surfer.nmr.mgh.harvard.edu). Information about how to download, install and use these softwares are available on the software websites. In addition, please see tutorials on how to run the retinotopy analysis (http://gru.stanford.edu/doku.php/mrtools/tutorialsretinotopy) and on how to run the pRF analysis (http://gru.stanford.edu/doku.php/mrtools/tutorialsprf) in mrTools. Humphrey visual fields metrics were obtained from the Humphrey STATPAC software (Zeiss Humphrey Systems) from the Humphrey Field Analyzer (HFA II 750), and then analyzed in Matlab (R2015b, https://www.mathworks.com/products/matlab.html).

For manuscripts utilizing custom algorithms or software that are central to the research but not yet described in published literature, software must be made available to editors and reviewers. We strongly encourage code deposition in a community repository (e.g. GitHub). See the Nature Research guidelines for submitting code & software for further information.

Data

Policy information about availability of data

All manuscripts must include a data availability statement. This statement should provide the following information, where applicable:

- Accession codes, unique identifiers, or web links for publicly available datasets
- A list of figures that have associated raw data
- A description of any restrictions on data availability

All processed data reported in this paper have been deposited in a database and are publicly available as of the date of publication at ADDLINK. Source data associated with this paper are also provided in the public repository. Anonymised raw data and additional information are available from the corresponding author upon reasonable request.

Field-specific reporting

Please select the one below that is the best fit for your research. If you are not sure, read the appropriate sections before making your selection.

[x] Life sciences [ ] Behavioural & social sciences [ ] Ecological, evolutionary & environmental sciences

For a reference copy of the document with all sections, see nature.com/documents/nr-reporting-summary-flat.pdf

Life sciences study design

All studies must disclose on these points even when the disclosure is negative.

Sample size	CB participants were recruited for visual training studies that have been already published (e.g., Huxlin et al., 2009; Das et al., 2014, Cavanaugh et al., 2015; Cavanaugh & Huxlin, 2017). The numbers of patients selected were driven by the numbers of patients who matched our inclusion criteria and who successfully completed those visual training studies, rather than by sample size calculations. The sample size of 11 CB patients (and 9 visually-intact controls) is substantially larger than most studies on cortical blindness, which often rely on single-cases or small patient groups.
Data exclusions	No data was excluded.
Replication	The experiment presented in the paper was repeated in multiple participants (N=11) and all results in the paper are drawn from the analysis of these multiple subjects. The effects were consistent across visual training regimes, which could vary between participants (see Methods). In addition, retinotopic maps, the extent of the lesions and the regions-of-interest were drawn independently by 2 of the authors (AD and AB); similar results were obtained. In a few problematic cases, MDM and EPM were also asked to independently draw these maps and regions of interest. Finally, as discussed in the paper, several of our results can be considered as replications of previous published studies (e.g., training-induced recovery of visual discrimination and visual field sensitivity, presence of spared V1 activity representing the blind field prior to training, coverage of blind-field regions by V1 population receptive fields prior to training).
Randomization	n/a. The same experimental protocol was used for all participant, and all findings are based on within-subject comparisons
Blinding	Blinding was not suitable for the present design, as all participants followed the same training regime. Data from each participant was analyzed through the same pipeline to ensure minimization of influence from the researcher.

Reporting for specific materials, systems and methods

We require information from authors about some types of materials, experimental systems and methods used in many studies. Here, indicate whether each material, system or method listed is relevant to your study. If you are not sure if a list item applies to your research, read the appropriate section before selecting a response.

Materials & experimental systems

n/a	Involved in the study	
[x]	[ ]	Antibodies
[x]	[ ]	Eukaryotic cell lines
[x]	[ ]	Palaeontology and archaeology
[x]	[ ]	Animals and other organisms
[ ]	[x]	Human research participants
[x]	[ ]	Clinical data
[x]	[ ]	Dual use research of concern

Methods

n/a	Involved in the study	
[x]	[ ]	ChIP-seq
[x]	[ ]	Flow cytometry
[ ]	[x]	MRI-based neuroimaging

Human research participants

Policy information about studies involving human research participants

Population characteristics	The present study tested eleven human adult participants (5 females; mean age: 61±13 years old). All participants suffered from large, contralesional, homonymous visual field defects, caused by stroke-induced damage to the occipital cortex. All participants underwent visual training starting at least 5 months after the stroke-induced occipital damage (35±68 months, ranging from 5 to 237 months).
Recruitment	We relied on stringent inclusion criteria and a uniform patient group, which allowed us to identify ubiquitous, functional changes mediating recovery in chronic, stroke-induced cortical blindness. We do not believe our recruitment criteria biased the findings of the present study. Our findings describe within-subject effects and all participants followed the same experimental protocol.<br><br>Recruitement criteria were:<br>- all patients suffered from stroke-induced damage to the occipital cortex that resulted in cortical blindness<br>- stroke-induced occipital damage occurred at least 5 months before the pre-training session, in order to eliminate spontaneous visual recovery generally occurring during the first 3 months following an occipital stroke<br>- patients had no ocular health problems, neurological and/or cognitive impairments, and none of them suffered from visual or other forms of neglect between the pre-training and post-training sessions<br>- all participants could maintain stable fixation during testing<br>- all patients trained for several weeks at home and showed visual recovery that could be verified in lab using controlled experimental settings<br>- all participants must be MRI-compatible, at least at the moment of the pre-training session
Ethics oversight	The Research Subjects Review Board at the University of Rochester Medical Center

Note that full information on the approval of the study protocol must also be provided in the manuscript.

Magnetic resonance imaging

Experimental design

Design type	block design
Design specifications	The retinotopic fMRI sequence was the same for all subjects, and identical for both pre- and post-training sessions. Each session lasted ~1.5h and consisted of:<br>- 1 in-plane T1 weighted (MPRAGE, magnetization prepared rapid gradient echo; 0.75x0.75x3mm) anatomical volume<br>- 3 high-resolution, T1-weighted anatomical volumes (MPRAGE, 1x1x1 mm)<br>- 6 runs of the wedge stimulus (3 clockwise and 3 counterclockwise), 10.5 cycles of 24s (168 volumes)<br>- 4 runs of the ring stimulus (2 expanding and 2 contracting), 10.5 cycles of 24s (168 volumes)<br>- 2 runs of coherent vs. incoherent motion (block alternation protocol, 18 s period), 11 cycles (176 volumes)
Behavioral performance measures	All subjects performed a task at fixation to minimize fixation breaks and to ensure consistent behavioral and attentional state throughout fMRI data acquisition. A two-interval forced-choice (2-IFC) luminance decrement detection task was performed with respect to fixation. A cyan cross was displayed at the beginning of each trial and was briefly dimmed during each interval. The two target intervals were separated by 500ms, during which the cross' color changed back again to cyan. After a final 500ms, the cross turned yellow signaling the subject should report which of the two intervals contained the dimmer cyan cross by pressing one of 2 buttons. Target luminance decrement was adjusted throughout each scanning session using a 2-down/1-up staircase to maintain performance near 71%-correct and maintain attention at fixation during both pre-training (mean: 71±9%-correct) and post-training (mean: 73±4%-correct) scanning sessions.

Acquisition

Imaging type(s)	functional and structural
Field strength	3 Tesla
Sequence & imaging parameters	Functional scans were acquired with gradient recalled echo-planar imaging to measure blood oxygen level-dependent (BOLD) changes in image intensity: 21 slices oriented perpendicular to the calcarine sulcus; repetition time 1.5s; echo time 30-ms; flip angle 75º; voxel size 3x3x3 mm; grid size 64x64. The retinotopic mapping area was constrained by the height of the visual display (11.5 degree radius from the center of the screen). Three high-resolution, T1-weighted anatomical volumes of the entire brain were collected for each subject at a resolution of 1mm isotropic at the end of the functional scanning session.
Area of acquisition	Occipital lobe (21 slices centered on the occipital cortex, and oriented perpendicular to the calcarine sulcus)

Diffusion MRI ☐ Used ☒ Not used

Preprocessing

Preprocessing software	mrTools and FreeSurfer. See above for links to software versions and tutorials.
Normalization	Data was not normalized as data was always analyzed in single-subject space. For each patient, three high-resolution, T1-weighted anatomical volumes were averaged together, and the average was used to extract and computationally flatten the cortical surface using FreeSurfer. In-plane, anatomical images were then aligned with the average high-resolution anatomical volume by an automated, robust image registration algorithm using mrTools software. The alignment parameters were used to project the measured fMRI responses onto the flattened cortical surfaces for each hemisphere and for each participant.
Normalization template	n/a. Data was not normalized as each participant was analyzed separately
Noise and artifact removal	Data from each run were preprocessed in mrTools using standard procedures for motion compensation and detrending.
Volume censoring	All MRI frames were included; volume censoring was not performed

Statistical modeling & inference

Model type and settings	Repeated-measures ANOVAs were used to assess the effects of within-subject factors. Generalized linear mixed-effects models with participants as a random effect were used for the regression analyses.
Effect(s) tested	Repeated-measures ANOVAs were used to test the effects of training (pre-training vs post-training) and visual-field coverage (intact-field vs blind-field) on visual dicrimination performance at trained, blind-field locations (Fig.1), as well as on population receptive fields (pRFs) estimates (Fig.7). Post-hoc tests were subsequently ran (when appropriate). In all cases in which the Mauchly's test of sphericity indicated a violation of the sphericity assumption, Greenhouse-Geisser corrected values were used. Partial eta-squared and Cohen's d values were calculated to assess effect size for ANOVAs and paired-sample t-tests, respectively. Wilcoxon signed-rank tests were used when the normality assumption was violated, with the effect size reported as the rank-biserial correlation (r). Generalized linear mixed-effects models, with participants as a random effect, were used to test whether pre-training variables (e.g., pre-training, visually-evoked response coherence of V1 voxels) can predict the magnitude of post-training visual recovery within the blind field of trained CB patients (e.g., Fig.4). The same regression approach was used to assess the effects of training (pre-training vs post-training) on V1 visually-evoked responses as a function of the magnitude of post-training visual recovery (Fig.5). A linear mixed-model regression model (with participants as a random effect) was also used to assess the effects of training and visual-field coverage on pRF size as a function of pRF eccentricity (Fig.7 and Fig.S8). Pearson's correlation analyses were also performed to test the relationship between different variables (e.g., Fig.7). More details can be found in the Results and Methods–Statistical analyses sections.

Specify type of analysis: ☐ Whole brain ☒ ROI-based ☐ Both

Anatomical location(s)	Primary visual cortex (V1); Extrastriate areas (V2, V3, V4)
Statistic type for inference (See Eklund et al. 2016)	voxel-wise
Correction	ANOVAs were followed by planned post-hoc comparisons (uncorrected), when appropriate

Models & analysis

n/a	Involved in the study
☒	☐ Functional and/or effective connectivity
☒	☐ Graph analysis
☒	☐ Multivariate modeling or predictive analysis
